# Building a predator: macroevolutionary patterns in the skull of abelisaurid dinosaurs

**DOI:** 10.1098/rspb.2025.0943

**Published:** 2025-11-05

**Authors:** Enzo E. S. Pereyra, Martín D. Ezcurra, Carolina Paschetta, Ariel H. Méndez

**Affiliations:** ^1^ Instituto Patagónico de Geología y Paleontología (CCT CONICET-CENPAT), Puerto Madryn, Chubut, Argentina; ^2^ Sección Paleontología de Vertebrados, CONICET-Museo Argentino de Ciencias Naturales ‘Bernardino Rivadavia’, Ciudad Autónoma de Buenos Aires, Argentina; ^3^ School of Geography, Earth, and Environmental Sciences, University of Birmingham, Birmingham, UK; ^4^ Instituto Patagónico de Ciencias Sociales y Humanas ‘Dra María Florencia del Castillo Bernal’ (CCT CONICET-CENPAT), Puerto Madryn, Chubut, Argentina; ^5^ Programa de Referencia y Biobanco Genómico de la Población Argentina, Argentina

**Keywords:** Abelisauridae, modularity, integration, evolutionary rate, disparity, cranial regions, skull

## Abstract

Abelisauridae is the most abundant and well-known clade of Gondwanan theropods, characterized by uniquely tall, robust and heavily ornamented skulls. Different processes have been proposed to explain the evolution of the abelisaurid skull based on anatomical and biomechanical studies, but a quantitative analysis under an explicit macroevolutionary approach is lacking. In this study, we assess patterns of modularity, integration and macroevolutionary trends of the abelisaurid skull by quantifying different cranial regions and bones using two-dimensional geometric morphometrics. Our results reveal phylogenetic modularity and evolutionary integration between the neurocranium and rostral regions. High disparity and evolutionary rates are found in the occipital region, squamosal, quadratojugal, lacrimal and postorbital. These findings suggest that the neurocranium was the main region involved in the proportional cranial height increase in Abelisauridae. The observed patterns are linked to different feeding strategies, supporting ecological specialization within this theropod lineage. We propose that ecological pressures were the main drivers of skull evolution in abelisaurids, with some features co-opted for socio-sexual display. Future research should focus on evolutionary modelling to investigate patterns of evolutionary rate selection and constraints that can explain the diversification of the skull shape of this clade during the Cretaceous.

## 1. Introduction

The evolution of different structures in an organism involves different degrees of association among traits [1]. Morphological integration refers to the strength and patterns of correlation between traits [1–4]. On the other hand, modularity refers to morphological complexes that evolve relatively independently from each other [2,5]. The effect of trait integration and modularity in a macroevolutionary framework can be manifested as differences in evolutionary trends and/or constraints [2]. Trait correlation regulates the axes of variation in which an evolutionary process can act [1].

Patterns of morphological integration and modularity have been investigated in the archosaur cranium with multivariate phenotypic approaches [1,6,7]. In particular, in non-avian dinosaurs, Felice *et al.* [1] used three-dimensional geometric morphometrics to recover modular signals and high degrees of evolutionary correlation among different cranial regions. In particular, the occipital region has been found to be highly integrated through the evolution of Archosauria. On the other hand, Marugán-Lobón & Buscalioni [6] proposed that the rostrum, orbit and braincase are independent evolving modules in Dinosauria. However, no study has focused on the cranial phylogenetic integration and modularity of deeper theropod dinosaur lineages. Non-avian theropods were morphologically [8–11] and ecologically very diverse [12,13]. Thus, studying the phylogenetic integration and modularity of cranial regions of clades within Theropoda could help us understand how phenotypic trends are associated with different ecological and/or socio-sexual selection pressures.

**Figure 1.**
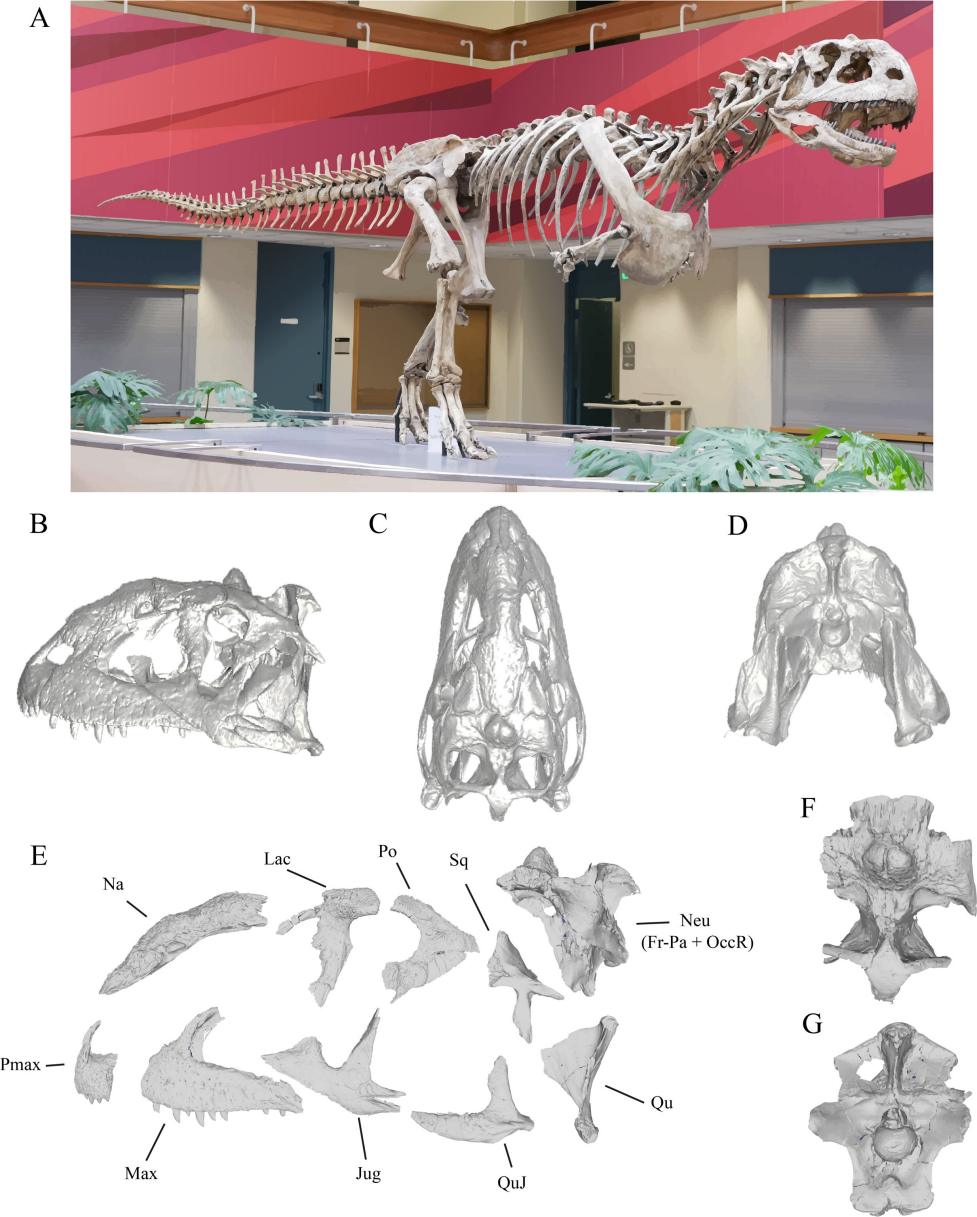
*Majungasaurus crenatissimus* (A) mounted skeleton at Stony Brook University (CC BY-ND) and (B–G) its skull. Skull in left lateral (B), dorsal (C), and posterior (D) views (taken from Sampson & Witmer [12]; https://people.ohio.edu/witmerl/Majungasaurus-skull03.htm). (E) Cranial regions used in the analyses of phylogenetic modularity, integration, disparity and evolutionary rates in left lateral view (taken from [12]). (F) Frontal-parietal in dorsal view and (G) occipital region in posterior view. Abbreviations: Pmax, premaxilla; Max, maxilla; Na, nasal; Jug, jugal; QuJ, quadratojugal; Qu, quadrate; Sq, squamosal; Po, postorbital; Lac, lacrimal; Neu, neurocranium; Fr-Pa, frontal-parietal; OccR, occipital region.

Abelisauridae is the most abundant and best-known clade of theropod dinosaurs that inhabited the ancient continent of Gondwana from the Early Jurassic to the end of the Cretaceous [14–16]. This group possesses distinctive cranial and postcranial traits that set them apart from other theropod groups [17]. Their skull is generally tall, short and ornamented (figure 1), with a highly kinetic intramandibular articulation [12,18,19]. Biomechanical studies of the abelisaurid skull and vertebral column have led to propose a specialized feeding strategy as ambush predators [12,13], and that the cranial features are linked to socio-sexual display [12,20]. However, most of these palaeobiological hypotheses remain to be tested under quantitative macroevolutionary frameworks.

Here we use two-dimensional geometric morphometrics to quantify the shape of several skull elements and explore a series of macroevolutionary phenomena to shed light on the processes that shaped the abelisaurid skull morphology, including: (i) patterns of phylogenetic integration and modularity among phenotypic traits, which allow making inferences on the mechanisms that generate evolutionary change [1–4]; (ii) differences in morphological disparity through time, which could be related to different evolutionary processes [21]; and (iii) calculation of evolutionary rates, which describe the dynamics of phenotypic evolution [22–25].

## 2. Material and methodology

### (a) Phylogenetic hypothesis

Our methods use an explicit phylogenetic approach, and we chose the phylogenetic tree of Abelisauridae recently published by Pereyra *et al.* [26] for our analyses. This phylogenetic hypothesis was obtained after analysing the phylogenetic data matrix of Pol *et al.* [16] under maximum parsimony with implied weighting (*k* = 3−8), and the subsequent calculation of a global reduced strict consensus tree from all the MPTs found in the analyses with different *k* values. The resultant consensus tree was time-calibrated with the stochastic *cal3* function implemented in the R package paleotree v. 3.4.7 [27]. Each calculation, based on a different set of branch, extinction and sampling rates, retained 10 trees, which resulted in 780 time-calibrated trees. Finally, a tree with mean lengths for each branch was generated using the function *consensus.edges* of the phytools v. 2.3-0 package [28], and all zero-length branches were replaced with 0.1 million years to allow subsequent calculations (see [26] for more details).

### (b) Geometric morphometrics

The abelisaurid skull was subdivided into twelve regions: premaxilla, maxilla, nasal, postorbital, jugal, lacrimal, quadratojugal, quadrate, squamosal, occipital region, frontal-parietal and inferior mandible. The frontal and parietal bones were treated as a single structure because they are usually fused in Abelisauridae [12]. A strong integration of the occipital region was found as ancestral for Archosauria [1], and this region is strongly co-ossified or strongly sutured in Abelisauridae. Thus, we decided to consider the occipital region as a single region. The inferior mandible was also treated as a single region to simplify this structure and because we were more interested in analysing the relationships between the other skull regions.

All the regions were digitized in lateral view, excluding the frontal-parietal (dorsal view), quadrate (posterior view) and occipital regions (posterior view), by a single researcher (EESP) to minimize inter-observer error. The left side or half median elements was used, and images were mirrored when only the right side was available. We built two-dimensional morpho-geometric constellations of the seven skull regions using landmarks and semilandmarks digitized with TPS.dig v. 2.6.4 [29] onto photographs or figures from the bibliography (see more details and landmark positions in electronic supplementary material, figures S1–S3 and tables S1–S14). We imported the landmark coordinates into the R software environment (v. 4.4.3). A generalized Procrustes analysis (*gpagen* function of the geomorph v. 4.0.9 package [30]) was performed to remove the effects of orientation, location and size from each region. To maximize the abelisaurid sample in our study, when osteological elements were partially preserved (electronic supplementary material, table S1), we estimated landmark locations (*estimate.missing* function, geomorph v. 4.0.9 package [30]) only if the region had at least 50% of the landmarks and semilandmarks (see electronic supplementary material, table S1).

### (c) Phylogenetic modularity and integration analysis

We used the covariance ratio (CR) (*phylo.modularity* function) in the geomorph v. 4.0.9 package [30] to quantify the degree of covariation between cranial regions among five modularity hypotheses: (i) all regions are phylogenetically integrated; (ii) rostrum, in which rostral elements (premaxilla, nasal and maxilla) are phylogenetically integrated; (iii) superior mandible, in which premaxilla, maxilla, jugal, quadratojugal and quadrate are phylogenetically integrated; (iv) rostrum + orbit, in which the premaxilla, nasal, maxilla, jugal, postorbital and lacrimal are phylogenetically integrated; and (v) all regions are modular, in which all regions evolve independently from each other. These hypotheses are based on previous studies that examined different archosaurian lineages, providing evidence of strong phylogenetic integration in the rostrum, orbit and upper jaw [1,6,7,31], as well as signals of modularity [1] and putative correlated osteological changes in abelisaurid skull elements [12].

The covariance ratio is obtained from a covariance matrix of the traits, and the significance of the test is evaluated using a permutation procedure [25,32]. Subsequently, the *compare.CR* function (geomorph v. 4.0.9 package [30]) was used to evaluate which modular hypothesis had more support. This test was performed only for those regions digitalized in lateral view, thus excluding the occipital and frontal-parietal regions. We did not perform a modularity test in dorsal view because the only complete configuration is limited to that of *Majungasaurus*.

We performed pairwise comparisons between skull regions using partial least squares to quantify the degree of phylogenetic covariation under a Brownian motion evolutionary model with 10 000 interactions (function *phylo.integration*, geomorph v. 4.0.9 package [30]). The observed value (r-pls) is evaluated after permuting the data of each partition relative to the others [4]. Additionally, a multivariate effect size (*Z*) is estimated from the empirical sampling distribution. This statistic describes the strength of the sample effect [32]. We based our interpretations mainly on the *Z* values, rather than the significance of the test, because the degree of correlation depends on the number of landmarks and taxa used [25,32], and our taxon sampling is relatively low. For each pairwise comparison, we tested the influence of size in shape in a phylogenetic context (*procD.pgls* function, geomorph v. 4.0.9 package [30]). When the allometry explained more than 10% of the shape data, we removed its effect using the residuals of the phylogenetic regression (electronic supplementary material, table S18) because we aim to elucidate patterns of shape covariation between cranial regions and not allometric trends.

We used skulls with all the bones of interest preserved and articulated for the phylogenetic modularity analysis and isolated bones for the phylogenetic integration analysis.

### (d) Disparity through time analysis

A disparity-through-time plot (dtt) was performed to visualize patterns of shape changes within and among clades for all cranial regions in the abelisaurid phylogeny (*dtt* function, geiger v. 2.0.11 package [33]). We used 90% of the variation after a phylogenetic principal component analysis (Phylo-PCA) because the disparity analysis is focused more on ecological than phylogenetic signals [32]. In the dtt, the disparity is estimated for all taxa of the phylogeny and subsequently for each subclade. Relative disparity is obtained by dividing each subclade disparity value by the total disparity of the clade. Finally, we estimated the average relative disparities of all the subclades at that time [21].

### (e) Evolutionary rates

Evolutionary rates of cranial regions were estimated using the set of Procrustes coordinates (*compare.evol.rates* function, geomorph v. 4.0.9 package [30]) under a Brownian evolution model with 10 000 interactions and a permutational procedure. This approach uses the outer-product matrix between taxa differences in the morphospace after phylogenetic transformation (see [24] for more details). Subsequently, the net rate of shape evolution is calculated in the multi-phenotypic space.

The R scripts and input files to conduct the modularity, integration, evolutionary rates and disparity analyses can be found in the public repository Zenodo [34].

## 3. Results

### (a) Phylogenetic modularity and integration between cranial regions

Our phylogenetic modularity test found that the hypothesis in which all cranial regions evolved independently from each other has more support, with all regions being significantly modular (CR < 1, *p* < 0.001; electronic supplementary material, table S15).

**Figure 2.**
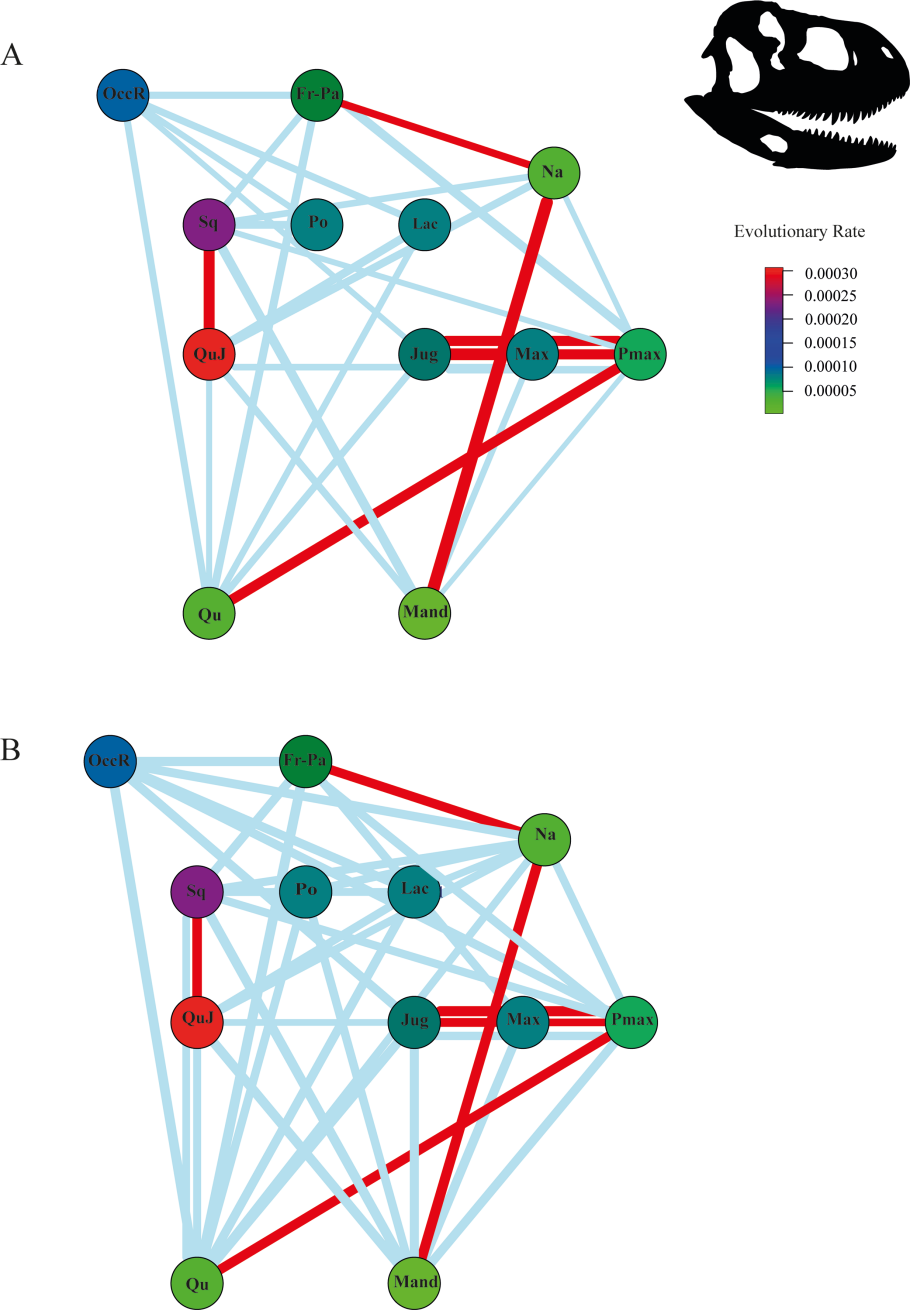
Network diagrams with evolutionary rates, as a colour thermometer, illustrating the (A) effect size values (Z) between cranial regions (>1), and the (B) results of the phylogenetic partial least squares (r-pls) between cranial regions (>0.75). Nodes represent cranial regions, and the thickness of the lines connecting nodes represents the strength of the covariation, in which red lines represent a significant correlation (*Skorpiovenator* silhouette taken from PhyloPic; http://phylopic.org). Abbreviations: Pmax, premaxilla; Max, maxilla; Na, nasal; Jug, jugal; QuJ, quadratojugal; Qu, quadrate; Sq, squamosal; Po, postorbital; Lac, lacrimal; Fr-Pa, frontal-parietal; Mand, inferior mandible.

Fifty-six phylogenetic pair comparisons were performed to assess the degree of covariation between cranial regions in Abelisauridae. The result showed (figure 2; electronic supplementary material, tables S16 and S17) a significant covariation between premaxilla and maxilla, maxilla and jugal, premaxilla and jugal, premaxilla and quadrate, frontal-parietal and nasal, squamosal and quadratojugal and inferior mandible and nasal. Additionally, other pairs of comparisons showed non-significant, but high covariation values: (i) the premaxilla with the nasal and squamosal; (ii) the quadrate was the element with higher phylogenetic covariation with all other skull elements except the maxilla, nasal and postorbital; (iii) elements that form the orbit (jugal, lacrimal and postorbital) strongly covaried with the occipital region, but weakly covaried among them; (iv) the occipital and the frontal-parietal regions; (v) the squamosal with the frontal-parietal, postorbital, quadratojugal, premaxilla and nasal; and (vi) the inferior mandible with the squamosal and cranial regions that form the superior mandible.

### (b) Disparity through time

**Figure 3.**
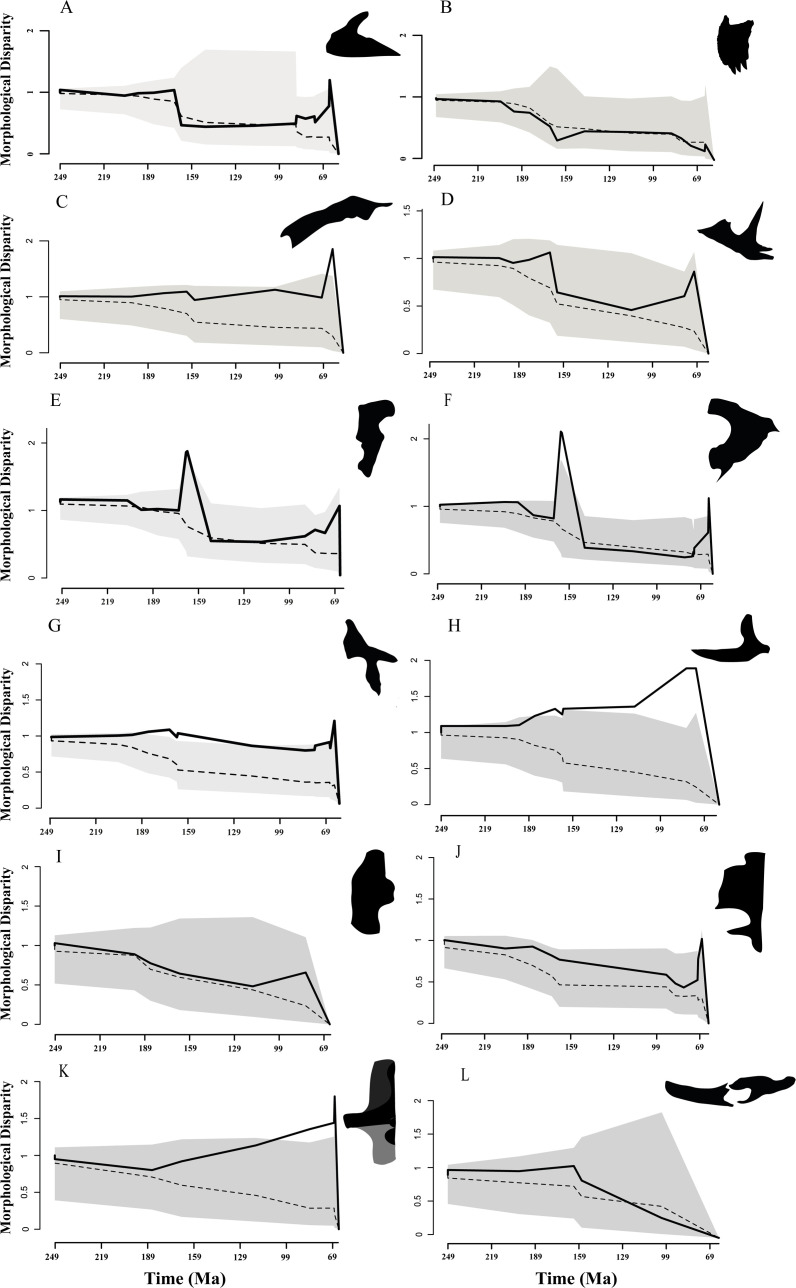
Morphological disparity through time estimated from 90% of the variance of the Phylo-PCs for the (A) maxilla, (B) premaxilla, (C) nasal, (D) jugal, (E) lacrimal, (F) postorbital, (G) squamosal, (H) quadratojugal, (I) quadrate and (J) frontal-parietal in dorsal views, (K) occipital region in posterior view and (L) inferior mandible in lateral view. The solid line represents the observed data, the dashed line represents the mean of simulations under the BM model, and the grey area is the 95% confidence interval of the simulated dataset (silhouettes created from skull osteological elements of *Majungasaurus crenatissimus* and *Carnotaurus sastrei*).

The dtt analysis showed a low morphological disparity between approximately 170 Ma and approximately 90 Ma (late Early Jurassic–early Late Cretaceous), but an increase between approximately 90 Ma and 66 Ma (late Upper Cretaceous). This analysis suggests a Brownian motion model for the evolution of the maxilla (figure 3A). On the contrary, the premaxilla showed a low morphological disparity during the Jurassic (approx. 195 Ma) that slightly decreased towards the Late Cretaceous (figure 3B). The nasal (figure 2C) showed a slight increase of disparity during the Early Jurassic (approx. 189 Ma) and Early Cretaceous (approx. 140 Ma), reaching a peak in the Late Cretaceous, between approximately 70 Ma and 66 Ma, which is outside the 95% confidence interval of the Brownian motion model.

The jugal, postorbital and lacrimal (figure 3D–F) showed similar disparity patterns over time, reaching a peak during the Jurassic (between 170 Ma and 150 Ma) and a valley during the Early Cretaceous (between 140 Ma and 100 Ma). Subsequently, these cranial regions reach a second peak in the Late Cretaceous. The lacrimal and postorbital did not follow a Brownian motion evolutionary model during this interval.

The squamosal (figure 3G) showed relatively steady disparity levels until reaching a peak in the Late Cretaceous (approx. 70 Ma). During this interval, the squamosal did not follow a Brownian evolutionary model.

The quadratojugal (figure 3H) showed a pronounced increase of disparity during the Early Jurassic (approx. 189 Ma) and a second, more conspicuous increase to reach a global peak in the Late Cretaceous (between approx. 80 Ma and 70 Ma). The quadratojugal also failed to follow a Brownian evolutionary process.

The quadrate (figure 3I) showed a relatively continuous decrease in morphological disparity from the Early Jurassic (approx. 195 Ma) to the end of the Early Cretaceous (approx. 105 Ma). After this period, a pronounced increase produced a peak of disparity during the Late Cretaceous (approx. 80 Ma), and subsequently, there was a decline by the latest Cretaceous (between approx. 75 Ma and 66 Ma). The evolutionary model of the quadrate was within the 95% confidence interval of the Brownian motion model.

The neurocranial regions (frontal-parietal and occipital regions) showed different patterns of disparity over time. The frontal-parietal (figure 3J) showed a continuous decrease of disparity, with a low slope, from the Late Jurassic (approx. 170 Ma) to the Late Cretaceous (approx. 85 Ma). Subsequently, a very conspicuous increase started to lead to a peak in the latest Cretaceous (approx. 69 Ma). The evolution of the frontal-parietal followed a Brownian evolutionary process. On the other hand, the occipital region (figure 3K) showed a strong increase in disparity starting in the Early Jurassic (approx. 189 Ma) to finish in a global peak in the latest Cretaceous (approx. 70 Ma). This region failed to follow a Brownian evolutionary model.

The inferior mandible (figure 3L) showed a pronounced decrease in disparity from the Middle Jurassic (approx. 160 Ma) to the end of the Cretaceous (approx. 66 Ma).

### (c) Evolutionary rates

Evolutionary rates were estimated to elucidate the evolutionary dynamics of each cranial region. The estimated evolutionary rates under Brownian evolutionary models showed that the quadratojugal, squamosal and the occipital region had the highest evolutionary rates of the cranial regions, followed by the lacrimal, postorbital and jugal (figure 2). Rostral elements, quadrate, inferior mandible and the frontal-parietal showed the lowest evolutionary rates.

## 4. Discussion

Our analyses of phylogenetic modularity, phylogenetic integration and evolutionary rates yielded results consistent with previous findings in Archosauria and non-avian dinosaurs, particularly regarding cranial modularity [1,31]. However, our study also revealed distinct patterns of skull evolutionary covariation and evolutionary rates in Abelisauridae that diverge from the broader archosaurian pattern.

Felice *et al.* [1] identified a high covariation between the quadrate, jugal and quadratojugal in non-avian dinosaurs. By contrast, our results indicate that, in abelisaurids, the premaxilla possesses a high phylogenetic covariation with the quadrate and a significant covariation with the maxilla. Additionally, the maxilla shows a significant phylogenetic covariation with the jugal and a high covariation with the quadratojugal. These findings suggest that the upper jaw followed similar macroevolutionary trends throughout the evolutionary history of the abelisaurids. The evolutionary rates of these regions reflect patterns observed in non-avian dinosaurs [31], with low rates detected in the premaxilla, maxilla and nasal. This indicates that the rostrum of abelisaurids maintained a high degree of morphological conservatism throughout their evolutionary history. Conversely, we observed high evolutionary rates and high levels of disparity for the quadratojugal and squamosal during the Late Cretaceous, with both regions being significantly phylogenetically integrated. High evolutionary rates have been previously found for this region in non-avian dinosaurs and linked to the evolution of cranial ornamentation and feeding adaptations [31].

One potential factor influencing rapid evolutionary changes in the squamosal and quadratojugal is their involvement in the jaw adductor chamber. The squamosal defines the boundaries of the superior temporal fenestra, which is the opening that allows the passage of key jaw-closing muscles to their origin areas on the skull roof [1,31]. As a result, changes in the shape of these bones likely influenced the biomechanics of prey capture and feeding efficiency in abelisaurids. Likewise, the quadratojugal plays an integral role in feeding mechanics. Its dorsoventral development contributes to the overall height of the adductor chamber, affecting the functional properties of the associated jaw musculature [31,35]. Anatomical and biomechanical studies of the skull and vertebral column, particularly of the cervical region, suggest that Late Cretaceous abelisaurids used a specialized predatory strategy based on ambush hunting [12,13,19,36]. This strategy probably involved using the head as a primary weapon, executing short-distance sprints and holding prey during the kill. Thus, evolutionary modifications in the squamosal and quadratojugal may have been strongly related to changes in bite force and prey handling in abelisaurids, potentially enhancing their ambush predation strategy.

We detected low evolutionary rates in the quadrate and frontal-parietal, contrasting with the results of Felice *et al.* [31]. Two potential explanations exist for this discrepancy: first, our use of two-dimensional landmark digitization may not have fully captured shape variation in these cranial regions, particularly those associated with the craniomandibular joint. Alternatively, these structures could have been highly conserved in Abelisauridae, possibly subjected to strong stabilizing selection. The latter hypothesis is supported by the low disparity values at the end of the Late Cretaceous and the consistently low evolutionary rates in these regions. Future studies employing three-dimensional geometric morphometrics will be necessary to determine whether our findings reflect a genuine macroevolutionary trend in Abelisauridae or an artefact of landmark digitization methodology.

Cranial regions forming the orbit (jugal, lacrimal and postorbital) were not evolutionarily integrated among them as Marugán-Lobón & Buscalioni [6] found in Dinosauria. However, in our analyses, these cranial regions strongly covaried with the occipital. Additionally, we detected high evolutionary rates in the postorbital, lacrimal and occipital regions, alongside an increase in disparity during the Late Cretaceous. The lacrimal and postorbital are hypermineralized structures that contribute to the formation of the orbit (e.g. [12,20]). The characteristic elliptical and keyhole-shaped orbits of the abelisaurids likely played a functional role in mitigating and dissipating stress during biting [37–40] and protecting the eye from potential blows incurred during intraspecific competition [20,41]. The bones that form the occipital region in Abelisauridae are co-ossified and strongly dorsally expanded, featuring a prominent nuchal crest [12,15]. This structure served as a large attachment site for muscles involved in head stabilization [12]. Thus, abelisaurids could have supported (i) torsional bending stress during the bite [12,36] and (ii) low-motion headbutting with conspecifics [20]. The observed macroevolutionary patterns and phylogenetic correlation results support the hypothesis that cranial modifications in Abelisauridae were functionally linked to both their feeding mechanisms and socio-sexual selection.

The high evolutionary rates in the occipital region, the low evolutionary rates in the rostrum and lower jaw and the significant evolutionary integration between the frontal-parietal and nasal regions suggest that the neurocranium was the main driver of cranial height increase in Abelisauridae. Hence, the dorsal expansion of the occipital region probably produced an increase of cranial height without curtailment of the other cranial regions that exhibit a high degree of phylogenetic covariation. This hypothesis is further supported by ontogenetic trajectories observed in *Majungasaurus* [42], in which skull height increases through ontogeny. Additionally, our dtt results indicate that the occipital region deviates from a Brownian motion model of evolution and increased morphological disparity over time. This pattern supports the hypothesis that ecological pressures associated with feeding mechanisms acted as a major evolutionary driver of the occipital region in Abelisauridae.

The inference of feeding behaviour and socio-sexual selection is based on the following morphological traits of Abelisauridae [12,19,20,36,37,39–41,43]: (i) an abbreviated skull that facilitates stabilization of the head using neck musculature, (ii) an increased bite force at the tip of the rostrum, (iii) a broad skull that resisted torsional bendings, (iv) a key-holed orbit shape that dissipated the tensions during the bite, (v) high bite force and (vi) great cursorial abilities. These characteristics suggest that the feeding mechanism primarily drove cranial modifications in Abelisauridae, with these adaptations later being co-opted for socio-sexual display, particularly with the emergence of hypermineralized cranial structures in Cretaceous abelisaurids, which possibly facilitated intraspecific combat. Here, we refer to this hypothesis as ‘feeding before display’ hypothesis. This hypothesis is supported by: (i) the functional role of elliptical orbits in dissipating feeding-induced stress without additional reinforcement of cranial bone structures [39], a feature that aligns with our findings of rapid evolution in this cranial region; (ii) the fact that early abelisaurids such as *Eoabelisaurus* (Early Jurassic) and *Spectrovenator* (Early Cretaceous) possess poorly developed skull roof structures compared with Late Cretaceous abelisaurids [44–46]; and (iii) the high evolutionary rates observed in the occipital region, squamosal and quadratojugal cranial regions, which are involved in bite force performance and head movement. The emergence of the specialized feeding strategy in later abelisaurids could have allowed these species to flourish during the Late Cretaceous.

The skull of Abelisauridae generally shows low disparity levels during the Late Jurassic and Early Cretaceous. However, it is important to note that the abelisaurid fossil record is scarce for this time interval. Therefore, a fossil sampling bias could influence the observed disparity patterns rather than reflecting a true biological trend. Future discoveries will help determine whether our findings represent a genuine pattern in the skull evolution of Abelisauridae. On the other hand, most cranial regions exhibit a strong disparity increase after approximately 90 Ma in the Late Cretaceous, reaching a peak before the K–Pg mass extinction. This change in the evolutionary dynamics of the abelisaurids matches with Cenomanian–Turonian faunal turnover, which involved the extinction of the carcharodontosaurid theropods and rebbachisaurid sauropods [17]. Fossil evidence suggests that abelisaurids, along with megaraptorans, diversified and occupied the ecological niches left vacant by the extinct carcharodontosaurids [11,17,47]. Our findings in cranial morphology support this hypothesis, suggesting that abelisaurids diversified after replacing carcharodontosaurids in their ecological roles. Future studies should investigate niche overlap between carcharodontosaurids and abelisaurids using species distribution modelling, integrating palaeoclimate data to assess their spatial distribution in the Cretaceous ecosystem.

Regarding specific cranial regions, the nasal, quadratojugal, squamosal, occipital region and lacrimal fall outside the 95% confidence interval expected under a Brownian motion model in the dtt analyses. This deviation suggests that these structures may have evolved under alternative evolutionary dynamics. One plausible scenario is an Ornstein–Uhlenbeck (OU) process, where evolution is guided towards one or more adaptive optima [22]. In this context, the observed patterns of cranial modularity and integration point to distinct selective regimes shaping these regions, potentially linked to ecological specializations or functional constraints that emerged during the Early or Late Cretaceous. Therefore, future research should employ a comprehensive evolutionary modelling framework to assess the fit of these alternative models and identify potential shifts in selective regimes across the phylogeny. The tempo and mode of these shifts will help clarify when these cranial regions began to diverge from background evolutionary expectations. Notably, a recent study on the abelisaurid maxilla [26] suggests that these structures experienced a rapid evolution in Cretaceous abelisaurids, and the specialization for predation in this clade may have originated earlier than previously thought. Thus, integrating morphological, ecological and phylogenetic data is needed to better understand the evolutionary forces shaping abelisaurid cranial architecture.

## 5. Conclusion

Our findings enlighten the trends that occurred in the skull evolution of Abelisauridae. Despite the modular nature of cranial regions in abelisaurids, we highlight that these regions were phylogenetically inegrated and exhibited high evolutionary rates in specific areas that did not follow the same evolutionary patterns observed in other archosaur lineages. Feeding specialization was probably the main driver of skull evolution in Abelisauridae, with certain cranial features later being co-opted for socio-sexual display. However, some cranial regions did not follow a Brownian motion model, the most common model used to infer macroevolutionary trends in multivariate phenotypes. To further investigate the evolution of each cranial region in Abelisauridae, future research should incorporate advanced evolutionary modelling approaches, such as Early Burst models, Ornstein–Uhlenbeck models with multiple phenotypic optima, and multi-rate Brownian motion models. Additionally, we could not directly assess whether the occipital and frontal-parietal regions functioned as modular structures compared with other cranial regions. This limitation stems from the high degree of co-ossification within these regions and the use of a two-dimensional geometric morphometric approach. However, given that the lateral cranial regions show a strong degree of modularity, we expect the occipital and frontal-parietal regions to follow a similar trend. Future studies should apply a three-dimensional geometric morphometric approach to further test our findings and provide a more comprehensive understanding of the abelisaurid skull evolution.

Finally, species distribution modelling could clarify niche partitioning patterns among South American theropods and offer new insights into the coexistence of large and medium-sized theropod dinosaurs during the Cretaceous of South America.

## Data Availability

The electronic supplementary material contain (1) the species name, bibliographic source and cranial regions used in the analysis, (2) landmark protocol for each cranial region, and (3) r-pls, z-values and allometry values for cranial regions pairwise comparison. The R scripts to conduct the modularity, integration, evolutionary rates and disparity analyses and the tps files with the landmark coordinates can be found in the public Zenodo repository [34]. Supplementary material is available online [48].
